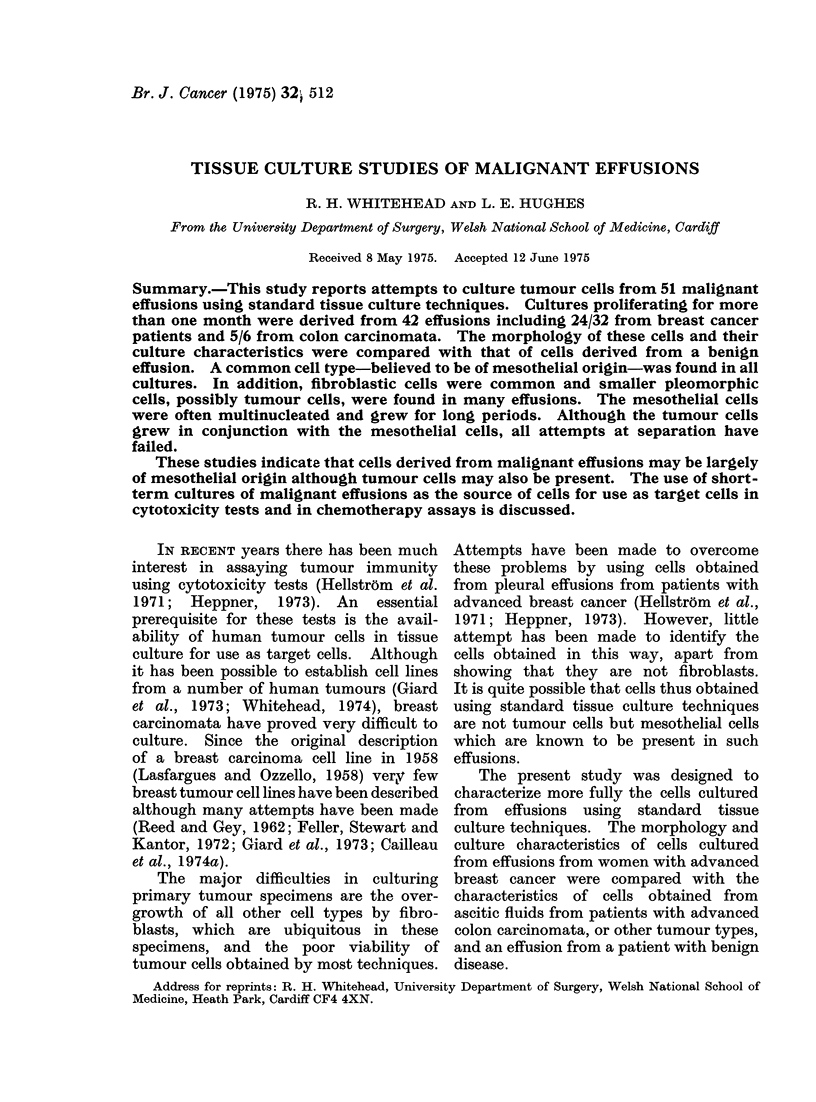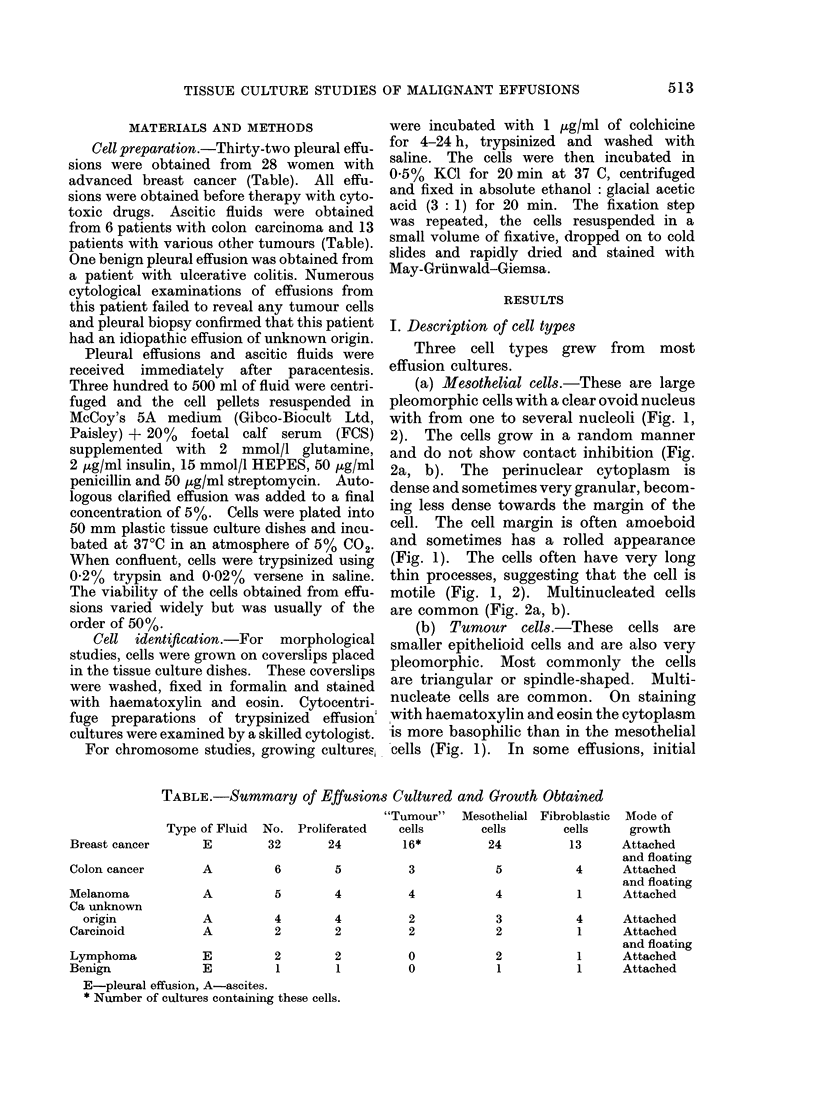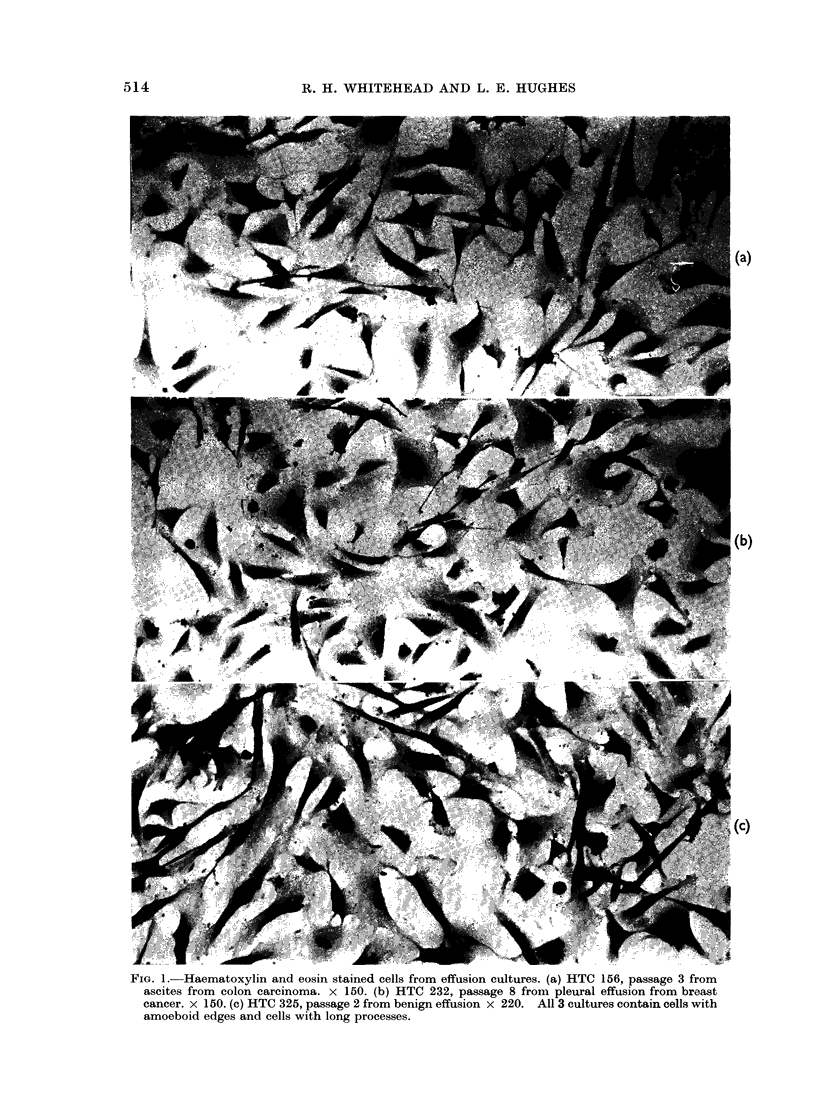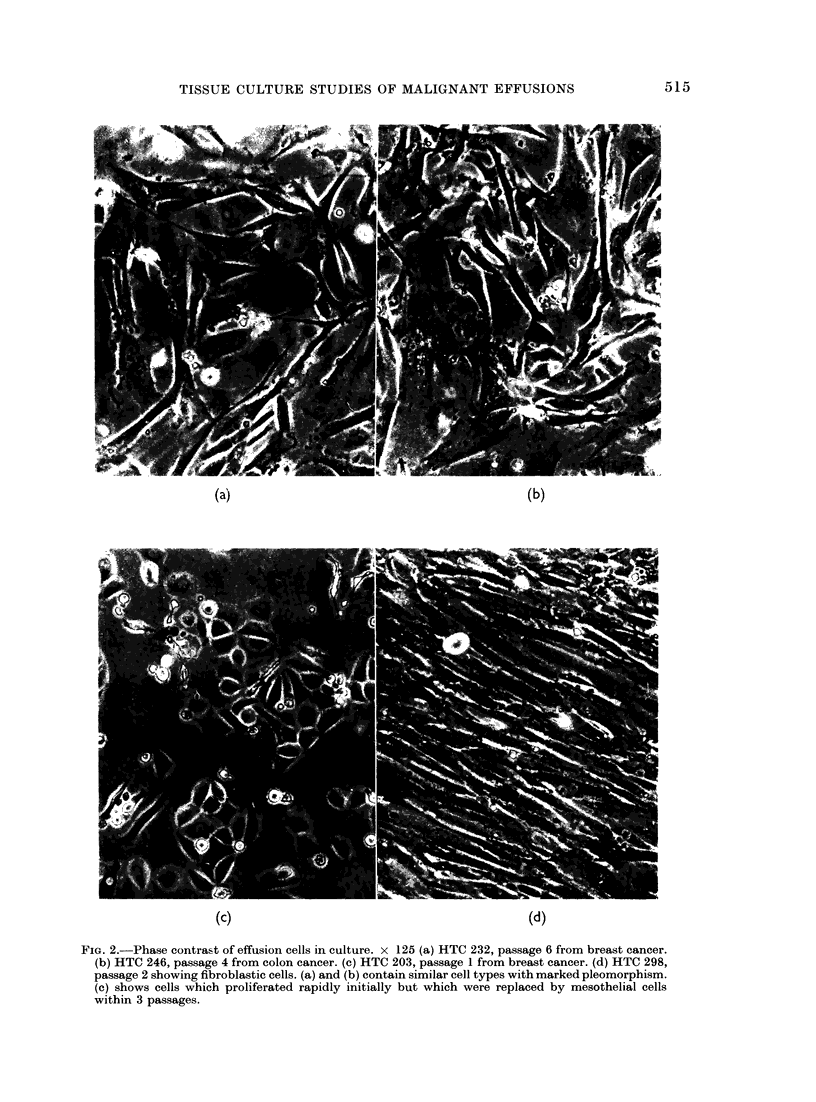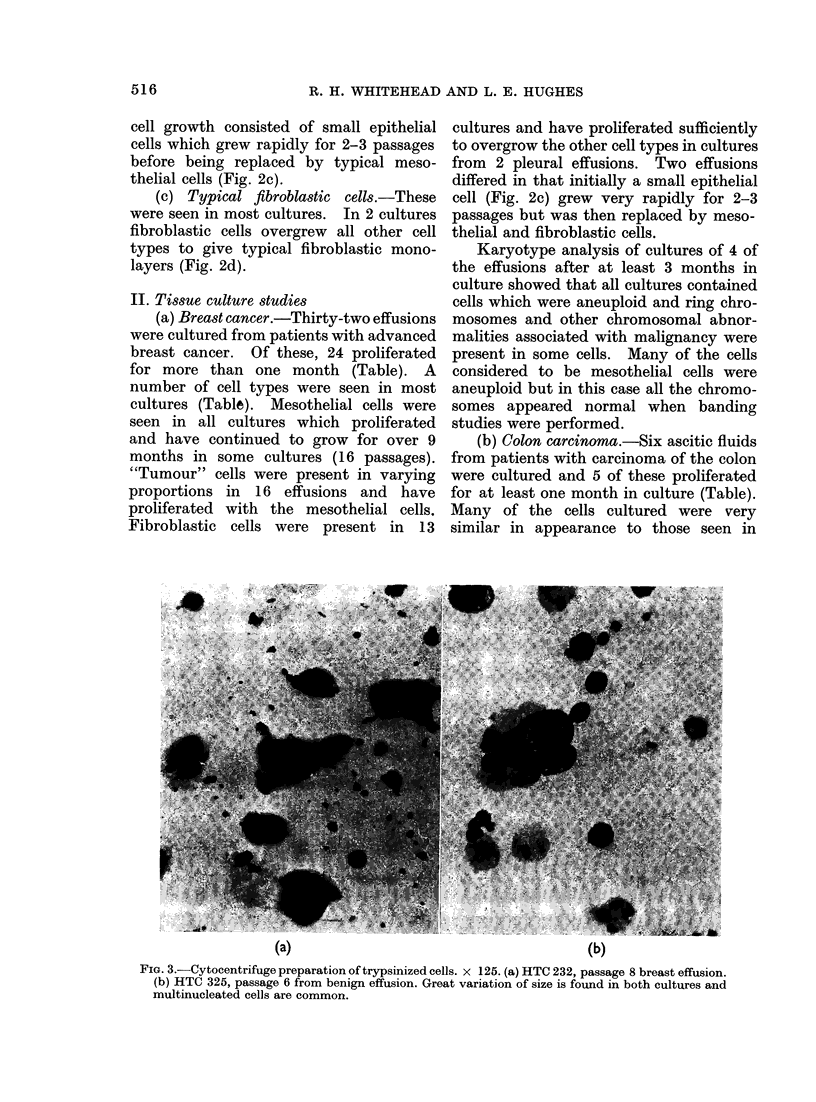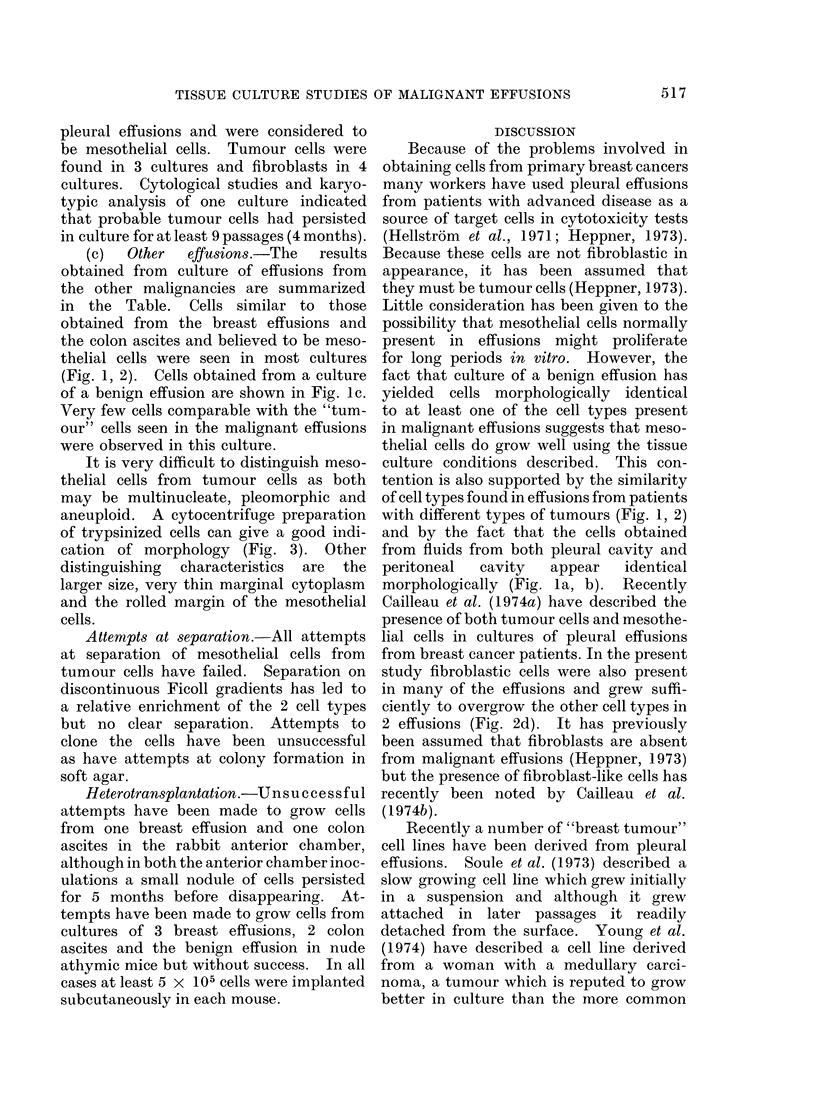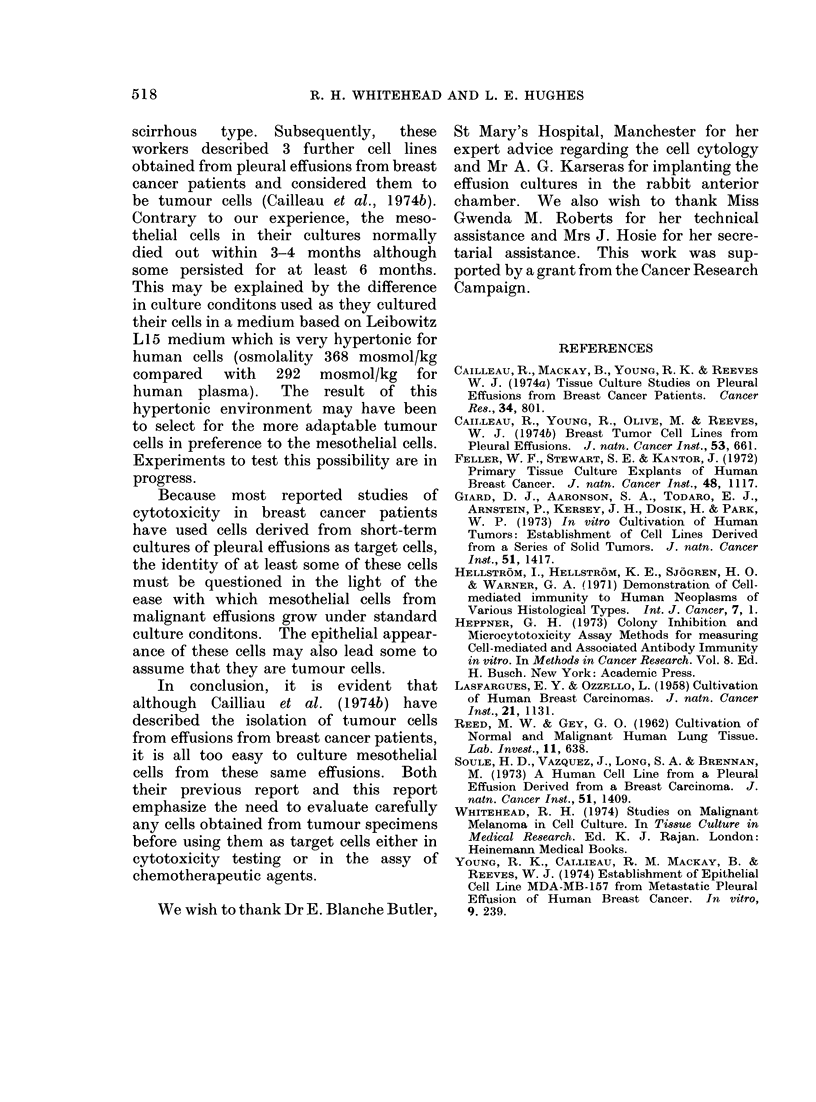# Tissue culture studies of malignant effusions.

**DOI:** 10.1038/bjc.1975.254

**Published:** 1975-10

**Authors:** R. H. Whitehead, L. E. Hughes

## Abstract

**Images:**


*Br. J. Cancer* (1975) **32**, 512

### TISSUE CULTURE STUDIES OF MALIGNANT EFFUSIONS

R. H. WHITEHEAD AND L. E. HUGHES

*From the University Department of Surgery, Welsh National School of Medicine, Cardiff*

Received 8 May 1975. Accepted 12 June 1975

**Summary.—This study reports attempts to culture tumour cells from 51 malignant effusions using standard tissue culture techniques. Cultures proliferating for more than one month were derived from 42 effusions including 24/32 from breast cancer patients and 5/6 from colon carcinomata. The morphology of these cells and their culture characteristics were compared with that of cells derived from a benign effusion. A common cell type—believed to be of mesothelial origin—was found in all cultures. In addition, fibroblastic cells were common and smaller pleomorphic cells, possibly tumour cells, were found in many effusions. The mesothelial cells were often multinucleated and grew for long periods. Although the tumour cells grew in conjunction with the mesothelial cells, all attempts at separation have failed.**

**These studies indicate that cells derived from malignant effusions may be largely of mesothelial origin although tumour cells may also be present. The use of short-term cultures of malignant effusions as the source of cells for use as target cells in cytotoxicity tests and in chemotherapy assays is discussed.**

In recent years there has been much interest in assaying tumour immunity using cytotoxicity tests (Hellström *et al.* 1971; Heppner, 1973). An essential prerequisite for these tests is the availability of human tumour cells in tissue culture for use as target cells. Although it has been possible to establish cell lines from a number of human tumours (Giard *et al.*, 1973; Whitehead, 1974), breast carcinomata have proved very difficult to culture. Since the original description of a breast carcinoma cell line in 1958 (Lasfargues and Ozzello, 1958) very few breast tumour cell lines have been described although many attempts have been made (Reed and Gey, 1962; Feller, Stewart and Kantor, 1972; Giard *et al.*, 1973; Cailleau *et al.*, 1974*a*).

The major difficulties in culturing primary tumour specimens are the overgrowth of all other cell types by fibroblasts, which are ubiquitous in these specimens, and the poor viability of tumour cells obtained by most techniques. Attempts have been made to overcome these problems by using cells obtained from pleural effusions from patients with advanced breast cancer (Hellström *et al.*, 1971; Heppner, 1973). However, little attempt has been made to identify the cells obtained in this way, apart from showing that they are not fibroblasts. It is quite possible that cells thus obtained using standard tissue culture techniques are not tumour cells but mesothelial cells which are known to be present in such effusions.

The present study was designed to characterize more fully the cells cultured from effusions using standard tissue culture techniques. The morphology and culture characteristics of cells cultured from effusions from women with advanced breast cancer were compared with the characteristics of cells obtained from ascitic fluids from patients with advanced colon carcinomata, or other tumour types, and an effusion from a patient with benign disease.

Address for reprints: R. H. Whitehead, University Department of Surgery, Welsh National School of Medicine, Heath Park, Cardiff CF4 4XN.

### MATERIALS AND METHODS

*Cell preparation.*—Thirty-two pleural effusions were obtained from 28 women with advanced breast cancer (Table). All effusions were obtained before therapy with cytotoxic drugs. Ascitic fluids were obtained from 6 patients with colon carcinoma and 13 patients with various other tumours (Table). One benign pleural effusion was obtained from a patient with ulcerative colitis. Numerous cytological examinations of effusions from this patient failed to reveal any tumour cells and pleural biopsy confirmed that this patient had an idiopathic effusion of unknown origin.

Pleural effusions and ascitic fluids were received immediately after paracentesis. Three hundred to 500 ml of fluid were centrifuged and the cell pellets resuspended in McCoy's 5A medium (Gibco-Biocult Ltd, Paisley) + 20% foetal calf serum (FCS) supplemented with 2 mmol/l glutamine, 2 μg/ml insulin, 15 mmol/l HEPES, 50 μg/ml penicillin and 50 μg/ml streptomycin. Autologous clarified effusion was added to a final concentration of 5%. Cells were plated into 50 mm plastic tissue culture dishes and incubated at 37°C in an atmosphere of 5% $CO_2$. When confluent, cells were trypsinized using 0·2% trypsin and 0·02% versene in saline. The viability of the cells obtained from effusions varied widely but was usually of the order of 50%.

*Cell identification.*—For morphological studies, cells were grown on coverslips placed in the tissue culture dishes. These coverslips were washed, fixed in formalin and stained with haematoxylin and eosin. Cytocentrifuge preparations of trypsinized effusion cultures were examined by a skilled cytologist.

For chromosome studies, growing cultures were incubated with 1 μg/ml of colchicine for 4–24 h, trypsinized and washed with saline. The cells were then incubated in 0·5% KCl for 20 min at 37 C, centrifuged and fixed in absolute ethanol : glacial acetic acid (3 : 1) for 20 min. The fixation step was repeated, the cells resuspended in a small volume of fixative, dropped on to cold slides and rapidly dried and stained with May-Grünwald–Giemsa.

### RESULTS

### I. *Description of cell types*

Three cell types grew from most effusion cultures.

(a) *Mesothelial cells.*—These are large pleomorphic cells with a clear ovoid nucleus with from one to several nucleoli (Fig. 1, 2). The cells grow in a random manner and do not show contact inhibition (Fig. 2a, b). The perinuclear cytoplasm is dense and sometimes very granular, becoming less dense towards the margin of the cell. The cell margin is often amoeboid and sometimes has a rolled appearance (Fig. 1). The cells often have very long thin processes, suggesting that the cell is motile (Fig. 1, 2). Multinucleated cells are common (Fig. 2a, b).

(b) *Tumour cells.*—These cells are smaller epithelioid cells and are also very pleomorphic. Most commonly the cells are triangular or spindle-shaped. Multinucleate cells are common. On staining with haematoxylin and eosin the cytoplasm is more basophilic than in the mesothelial cells (Fig. 1). In some effusions, initial

TABLE.—*Summary of Effusions Cultured and Growth Obtained*

| | Type of Fluid | No. | Proliferated | "Tumour" cells | Mesothelial cells | Fibroblastic cells | Mode of growth |
|---|---|---|---|---|---|---|---|
| Breast cancer | E | 32 | 24 | 16* | 24 | 13 | Attached and floating |
| Colon cancer | A | 6 | 5 | 3 | 5 | 4 | Attached and floating |
| Melanoma | A | 5 | 4 | 4 | 4 | 1 | Attached |
| Ca unknown origin | A | 4 | 4 | 2 | 3 | 4 | Attached |
| Carcinoid | A | 2 | 2 | 2 | 2 | 1 | Attached and floating |
| Lymphoma | E | 2 | 2 | 0 | 2 | 1 | Attached |
| Benign | E | 1 | 1 | 0 | 1 | 1 | Attached |

E—pleural effusion, A—ascites.
* Number of cultures containing these cells.

FIG. 1.—Haematoxylin and eosin stained cells from effusion cultures. (a) HTC 156, passage 3 from ascites from colon carcinoma. × 150. (b) HTC 232, passage 8 from pleural effusion from breast cancer. × 150. (c) HTC 325, passage 2 from benign effusion × 220. All 3 cultures contain cells with amoeboid edges and cells with long processes.

FIG. 2.—Phase contrast of effusion cells in culture. × 125 (a) HTC 232, passage 6 from breast cancer. (b) HTC 246, passage 4 from colon cancer. (c) HTC 203, passage 1 from breast cancer. (d) HTC 298, passage 2 showing fibroblastic cells. (a) and (b) contain similar cell types with marked pleomorphism. (c) shows cells which proliferated rapidly initially but which were replaced by mesothelial cells within 3 passages.

cell growth consisted of small epithelial cells which grew rapidly for 2–3 passages before being replaced by typical mesothelial cells (Fig. 2c).

(c) *Typical fibroblastic cells.*—These were seen in most cultures. In 2 cultures fibroblastic cells overgrew all other cell types to give typical fibroblastic monolayers (Fig. 2d).

II. *Tissue culture studies*

(a) *Breast cancer.*—Thirty-two effusions were cultured from patients with advanced breast cancer. Of these, 24 proliferated for more than one month (Table). A number of cell types were seen in most cultures (Table). Mesothelial cells were seen in all cultures which proliferated and have continued to grow for over 9 months in some cultures (16 passages). "Tumour" cells were present in varying proportions in 16 effusions and have proliferated with the mesothelial cells. Fibroblastic cells were present in 13 cultures and have proliferated sufficiently to overgrow the other cell types in cultures from 2 pleural effusions. Two effusions differed in that initially a small epithelial cell (Fig. 2c) grew very rapidly for 2–3 passages but was then replaced by mesothelial and fibroblastic cells.

Karyotype analysis of cultures of 4 of the effusions after at least 3 months in culture showed that all cultures contained cells which were aneuploid and ring chromosomes and other chromosomal abnormalities associated with malignancy were present in some cells. Many of the cells considered to be mesothelial cells were aneuploid but in this case all the chromosomes appeared normal when banding studies were performed.

(b) *Colon carcinoma.*—Six ascitic fluids from patients with carcinoma of the colon were cultured and 5 of these proliferated for at least one month in culture (Table). Many of the cells cultured were very similar in appearance to those seen in

FIG. 3.—Cytocentrifuge preparation of trypsinized cells. × 125. (a) HTC 232, passage 8 breast effusion. (b) HTC 325, passage 6 from benign effusion. Great variation of size is found in both cultures and multinucleated cells are common.

pleural effusions and were considered to be mesothelial cells. Tumour cells were found in 3 cultures and fibroblasts in 4 cultures. Cytological studies and karyotypic analysis of one culture indicated that probable tumour cells had persisted in culture for at least 9 passages (4 months).

(c) *Other effusions*.—The results obtained from culture of effusions from the other malignancies are summarized in the Table. Cells similar to those obtained from the breast effusions and the colon ascites and believed to be mesothelial cells were seen in most cultures (Fig. 1, 2). Cells obtained from a culture of a benign effusion are shown in Fig. 1c. Very few cells comparable with the "tumour" cells seen in the malignant effusions were observed in this culture.

It is very difficult to distinguish mesothelial cells from tumour cells as both may be multinucleate, pleomorphic and aneuploid. A cytocentrifuge preparation of trypsinized cells can give a good indication of morphology (Fig. 3). Other distinguishing characteristics are the larger size, very thin marginal cytoplasm and the rolled margin of the mesothelial cells.

*Attempts at separation*.—All attempts at separation of mesothelial cells from tumour cells have failed. Separation on discontinuous Ficoll gradients has led to a relative enrichment of the 2 cell types but no clear separation. Attempts to clone the cells have been unsuccessful as have attempts at colony formation in soft agar.

*Heterotransplantation*.—Unsuccessful attempts have been made to grow cells from one breast effusion and one colon ascites in the rabbit anterior chamber, although in both the anterior chamber inoculations a small nodule of cells persisted for 5 months before disappearing. Attempts have been made to grow cells from cultures of 3 breast effusions, 2 colon ascites and the benign effusion in nude athymic mice but without success. In all cases at least $5 \times 10^5$ cells were implanted subcutaneously in each mouse.

### DISCUSSION

Because of the problems involved in obtaining cells from primary breast cancers many workers have used pleural effusions from patients with advanced disease as a source of target cells in cytotoxicity tests (Hellström *et al.*, 1971; Heppner, 1973). Because these cells are not fibroblastic in appearance, it has been assumed that they must be tumour cells (Heppner, 1973). Little consideration has been given to the possibility that mesothelial cells normally present in effusions might proliferate for long periods *in vitro*. However, the fact that culture of a benign effusion has yielded cells morphologically identical to at least one of the cell types present in malignant effusions suggests that mesothelial cells do grow well using the tissue culture conditions described. This contention is also supported by the similarity of cell types found in effusions from patients with different types of tumours (Fig. 1, 2) and by the fact that the cells obtained from fluids from both pleural cavity and peritoneal cavity appear identical morphologically (Fig. 1a, b). Recently Cailleau *et al.* (1974*a*) have described the presence of both tumour cells and mesothelial cells in cultures of pleural effusions from breast cancer patients. In the present study fibroblastic cells were also present in many of the effusions and grew sufficiently to overgrow the other cell types in 2 effusions (Fig. 2d). It has previously been assumed that fibroblasts are absent from malignant effusions (Heppner, 1973) but the presence of fibroblast-like cells has recently been noted by Cailleau *et al.* (1974*b*).

Recently a number of "breast tumour" cell lines have been derived from pleural effusions. Soule *et al.* (1973) described a slow growing cell line which grew initially in a suspension and although it grew attached in later passages it readily detached from the surface. Young *et al.* (1974) have described a cell line derived from a woman with a medullary carcinoma, a tumour which is reputed to grow better in culture than the more common scirrhous type. Subsequently, these workers described 3 further cell lines obtained from pleural effusions from breast cancer patients and considered them to be tumour cells (Cailleau *et al.*, 1974*b*). Contrary to our experience, the mesothelial cells in their cultures normally died out within 3–4 months although some persisted for at least 6 months. This may be explained by the difference in culture conditons used as they cultured their cells in a medium based on Leibowitz L15 medium which is very hypertonic for human cells (osmolality 368 mosmol/kg compared with 292 mosmol/kg for human plasma). The result of this hypertonic environment may have been to select for the more adaptable tumour cells in preference to the mesothelial cells. Experiments to test this possibility are in progress.

Because most reported studies of cytotoxicity in breast cancer patients have used cells derived from short-term cultures of pleural effusions as target cells, the identity of at least some of these cells must be questioned in the light of the ease with which mesothelial cells from malignant effusions grow under standard culture conditons. The epithelial appearance of these cells may also lead some to assume that they are tumour cells.

In conclusion, it is evident that although Cailliau *et al.* (1974*b*) have described the isolation of tumour cells from effusions from breast cancer patients, it is all too easy to culture mesothelial cells from these same effusions. Both their previous report and this report emphasize the need to evaluate carefully any cells obtained from tumour specimens before using them as target cells either in cytotoxicity testing or in the assy of chemotherapeutic agents.

We wish to thank Dr E. Blanche Butler, St Mary's Hospital, Manchester for her expert advice regarding the cell cytology and Mr A. G. Karseras for implanting the effusion cultures in the rabbit anterior chamber. We also wish to thank Miss Gwenda M. Roberts for her technical assistance and Mrs J. Hosie for her secretarial assistance. This work was supported by a grant from the Cancer Research Campaign.

### REFERENCES

CAILLEAU, R., MACKAY, B., YOUNG, R. K. & REEVES W. J. (1974*a*) Tissue Culture Studies on Pleural Effusions from Breast Cancer Patients. *Cancer Res.*, **34**, 801.

CAILLEAU, R., YOUNG, R., OLIVE, M. & REEVES, W. J. (1974*b*) Breast Tumor Cell Lines from Pleural Effusions. *J. natn. Cancer Inst.*, **53**, 661.

FELLER, W. F., STEWART, S. E. & KANTOR, J. (1972) Primary Tissue Culture Explants of Human Breast Cancer. *J. natn. Cancer Inst.*, **48**, 1117.

GIARD, D. J., AARONSON, S. A., TODARO, E. J., ARNSTEIN, P., KERSEY, J. H., DOSIK, H. & PARK, W. P. (1973) *In vitro* Cultivation of Human Tumors: Establishment of Cell Lines Derived from a Series of Solid Tumors. *J. natn. Cancer Inst.*, **51**, 1417.

HELLSTRÖM, I., HELLSTRÖM, K. E., SJÖGREN, H. O. & WARNER, G. A. (1971) Demonstration of Cell-mediated immunity to Human Neoplasms of Various Histological Types. *Int. J. Cancer*, **7**, 1.

HEPPNER, G. H. (1973) Colony Inhibition and Microcytotoxicity Assay Methods for measuring Cell-mediated and Associated Antibody Immunity *in vitro*. In *Methods in Cancer Research*. Vol. 8. Ed. H. Busch. New York: Academic Press.

LASFARGUES, E. Y. & OZZELLO, L. (1958) Cultivation of Human Breast Carcinomas. *J. natn. Cancer Inst.*, **21**, 1131.

REED, M. W. & GEY, G. O. (1962) Cultivation of Normal and Malignant Human Lung Tissue. *Lab. Invest.*, **11**, 638.

SOULE, H. D., VAZQUEZ, J., LONG, S. A. & BRENNAN, M. (1973) A Human Cell Line from a Pleural Effusion Derived from a Breast Carcinoma. *J. natn. Cancer Inst.*, **51**, 1409.

WHITEHEAD, R. H. (1974) Studies on Malignant Melanoma in Cell Culture. In *Tissue Culture in Medical Research*. Ed. K. J. Rajan. London: Heinemann Medical Books.

YOUNG, R. K., CALLIEAU, R. M. MACKAY, B. & REEVES, W. J. (1974) Establishment of Epithelial Cell Line MDA-MB-157 from Metastatic Pleural Effusion of Human Breast Cancer. *In vitro*, **9**. 239.